# Kidney function, albuminuria, and their modification by genetic factors and risk of incident dementia in UK Biobank

**DOI:** 10.1186/s13195-023-01248-z

**Published:** 2023-08-21

**Authors:** Tian-Shin Yeh, Lei Clifton, Jennifer A. Collister, Xiaonan Liu, David J. Hunter, Thomas J. Littlejohns

**Affiliations:** 1 Department of Physical Medicine and Rehabilitation, School of Medicine, College of Medicine, Taipei Medical University, No.250, Wuxing St., Taipei, 11031 Taiwan; 2 Department of Physical Medicine and Rehabilitation, Wan Fang Hospital, Taipei Medical University, Taipei, Taiwan; 3 Nuffield Department of Population Health, University of Oxford, Oxford, UK; 4 Department of Epidemiology and Nutrition, Harvard T. H. Chan School of Public Health, Harvard University, Boston, MA USA; 5 Department of Physical Medicine and Rehabilitation, National Taiwan University Hospital, Taipei, Taiwan; 6 Department of Physical Medicine and Rehabilitation, College of Medicine, National Taiwan University, Taipei, Taiwan

**Keywords:** Kidney function, Estimated glomerular filtration rate, Albuminuria, Genetics, Polygenic risk, Dementia, Longitudinal, UK Biobank

## Abstract

**Background:**

Associations between kidney function and dementia risk are inconclusive. Chronic kidney disease (CKD) severity is determined by levels of both estimated glomerular filtration rate (eGFR) and the urine albumin to creatinine ratio (ACR). However, whether there is a graded increase in dementia risk for worse eGFR in each ACR category is unclear. Also, whether genetic risk for dementia impacts the associations is unknown. The current study aims to investigate the associations between eGFR and albuminuria with dementia risk both individually and jointly, whether the associations vary by different follow-up periods, and whether genetic factors modified the associations.

**Methods:**

In 202,702 participants aged ≥ 60 years from the UK Biobank, Cox proportional-hazards models were used to examine the associations between eGFR and urine albumin creatinine ratio (ACR) with risk of incident dementia. GFR was estimated based on serum creatinine, cystatin C, or both. The models were restricted to different follow-up periods (< 5 years, 5–10 years, and ≥ 10 years) to investigate potential reverse causation.

**Results:**

Over 15 years of follow-up, 6,042 participants developed dementia. Decreased kidney function (eGFR < 60 ml/min/1.73m^2^) was associated with an increased risk of dementia (Hazard Ratio [HR] = 1.42, 95% Confidence Interval [CI] 1.28–1.58), compared to normal kidney function (≥ 90 ml/min/1.73m^2^). The strength of the association remained consistent when the models were restricted to different periods of follow-up. The HRs for incident dementia were 1.16 (95% CI 1.07–1.26) and 2.24 (95% CI 1.79–2.80) for moderate (3-30 mg/mmol) and severely increased ACR (≥ 30 mg/mmol) compared to normal ACR (< 3 mg/mmol). Dose–response associations were observed when combining eGFR and ACR, with those in the severest eGFR and ACR group having the greatest risk of dementia (HR = 4.70, 95% CI 2.34–9.43). APOE status significantly modified the association (*p* = 0.04), with stronger associations observed among participants with a lower genetic risk of dementia. There was no evidence of an interaction between kidney function and non-APOE polygenic risk of dementia with dementia risk (*p* = 0.42).

**Conclusions:**

Kidney dysfunction and albuminuria were individually and jointly associated with higher dementia risk. The associations were greater amongst participants with a lower genetic risk of dementia based on APOE, but not non-APOE polygenic risk.

**Supplementary Information:**

The online version contains supplementary material available at 10.1186/s13195-023-01248-z.

## Introduction

Dementia and chronic kidney disease (CKD) are important global public health issues associated with increased mortality, morbidity, and substantial economic costs [1, 2]. The prevalence of both diseases continues to rise in the rapidly aging global population [3, 4]. This is cause for concern as emerging evidence suggests that CKD may be implicated in dementia risk [5, 6]. A range of health conditions affecting different systems, such as diabetes, hypertension, and depression, have been identified as key targets for dementia prevention [7], and kidney dysfunction could represent a novel modifiable risk factor for dementia.

The definition of CKD is based on several criteria. First, a persistent decrease in glomerular filtration rate (GFR), the best overall indicator of kidney function, of below 60 ml/min/1.73m^2^. Another is the presence of kidney damage such as albuminuria, which is typically evaluated by the urine albumin to creatinine ratio (ACR) [8]. GFR is most commonly estimated based on serum creatinine [9], however, creatinine levels can be influenced by muscle mass, diet, chronic illnesses, and certain medications [10]. Therefore, the use of both creatinine and cystatin C for GFR estimation has been suggested as the more accurate and precise method [9].

Although potential mechanisms for the link between kidney dysfunction and dementia have been proposed, including oxidative stress, vascular damage, and increased uremic neurotoxins [11, 12], epidemiologic studies on the association between kidney function and dementia have produced inconclusive findings [13], especially when GFR was estimated using different biomarkers [14–16]. Also, CKD severity is determined based on the extent of both GFR decrement and albuminuria [17], and a recent study (*n* = 9,967) found that increased CKD severity based on estimated GFR (eGFR) and ACR combined was associated with dementia risk [14]. However, it is necessary to replicate this finding in larger populations, especially as this study did not have an adequate sample size to investigate the associations in those with the most severe CKD. In addition, many previous studies have relatively short follow-up duration and might not be able to rule out reverse causation bias. Longer follow-up is necessary to account for the possibility that preclinical dementia is driving the observed associations and account for the delay in diagnosis due to a reliance on medical records. Moreover, some previous studies have suggested that the association between kidney function and dementia was attributed to stroke [16, 18]; however, two recent studies that have explored this possibility [16, 19] have yielded conflicting results. Furthermore, APOE ε4 is a well-known risk factor for Alzheimer’s disease and is also linked with better kidney function [20–22]. Conversely, APOE ε2 is associated with a lower risk of Alzheimer’s disease but poorer kidney function [20–22]. As well as APOE, recent genome-wide association studies have identified additional single nucleotide polymorphisms (SNPs) significantly associated with dementia risk [23]. ‘Polygenic risk scores’ (PRS) can be generated to represent the aggregated genetic effects of these SNPs [24] and are potentially powerful novel tools for disease prediction and risk stratification. Consequently, it is important to understand whether inherited predisposition to dementia modifies any observed association between kidney function and dementia risk to inform personalized dementia prevention; however, there is a paucity of literature investigating this.

In the current study, we investigated the association between eGFR and albuminuria with the risk of dementia over 15 years in a population-based cohort of more than 200,000 participants. We also investigated whether associations vary by different follow-up periods, whether genetic factors—both APOE and non-APOE polygenic risk—modified the associations, and whether the associations varied using serum creatinine, cystatin C, or both, as measures of kidney function.

## Methods

### UK Biobank

Participants were selected from UK Biobank, a population-based cohort of half a million women and men aged 40–69 years old recruited between 2006–10 [25]. All participants attended one of 22 baseline assessment centers located in England, Scotland, and Wales. At baseline assessment, participants provided sociodemographic, lifestyle, and health-related information via a touchscreen questionnaire and verbal interview, underwent a range of physical examinations, and provided biological samples. Longitudinal data is captured through ongoing linkage to cohort-wide electronic medical records, thatincludes hospital inpatient and death records. In the current study, we included only participants aged 60 years or over at baseline to ensure that the analytic sample was restricted to those most at risk of developing dementia during follow-up. We further excluded participants with prevalent dementia, prevalent end-stage kidney disease, and with missing data on the main exposure.

### Kidney function exposures

The sample collection and processing procedure in the UK Biobank have been described in detail elsewhere [26, 27]. Briefly, spot urine and blood samples were collected at baseline assessment centers and stored in two separate locations, one a -80° automated archive and the other a -180° manual liquid nitrogen archive. Urinary and serum creatinine were measured by an enzymatic method on a Beckman Coulter AU5400 clinical chemistry analyzer using the manufacturer’s reagents and calibrators [28, 29]. Serum cystatin C was measured using a latex enhanced immunoturbidimetric method using a Siemens Advia 1800^5^. Urinary microalbumin was measured by an immunoturbidimetric method using reagents and calibrators sourced from Randox Bioscience [28].

The primary exposure was eGFR based on both serum creatinine and cystatin C (eGFRcr-cys) derived using the equation developed by the Chronic Kidney Disease Epidemiology Collaboration (CKD-EPI) [9]. CKD-EPI equations were also used to derive the secondary exposures 1) eGFR estimated from serum creatinine (eGFRcr) and 2) eGFR estimated from cystatin C (eGFRcys). All eGFR exposures were initially categorized into the following groups using the Kidney Disease: Improving Global Outcomes (KDIGO) definition [17] of chronic kidney disease status: ≥ 90 (normal or high), 60–89 (mildly decreased) 45–59 (mildly to moderately decreased), 30–44 (moderately to severely decreased) and  < 30 (severely decreased) ml/min/1.73m^2^. The latter three groups had low numbers of participants so were combined into a single category of  < 60 ml/min/1.73m^2^.

An additional secondary exposure, urine ACR, was derived by dividing urinary microalbuminuria by urinary creatinine and categorized into  < 3 (normal to mildly increased), 3–30 (moderately increased), and  > 30 (severely increased) mg/mmol. To derive ACR for all participants, individuals with microalbumin below the detectable level were assigned a value of 6.7 mg/L (65% of participants), the minimum detectable level of the assay [28].

### Dementia outcome

Dementia was determined using hospital inpatient and death registry records. Hospital inpatient records were obtained from Hospital Episode Statistics (HES) for England, Scottish Morbidity Record for Scotland, and Patient Episode Database for Wales. Death registry records were obtained from NHS Digital for England and Wales and Information and Statistics Division for Scotland. Primary and secondary hospital diagnoses and underlying and contributory causes of death were recorded using the International Classification of Diseases (ICD) coding system. The ICD codes used to ascertain dementia cases in the current study were previously selected and validated by the UK Biobank outcome adjudication group and are provided in Table S1 [30].

### Covariates

Sociodemographic [31], lifestyle [32], and health-related characteristics [33] were identified as potential confounders in the association between kidney function and dementia and were used as covariates in the analysis. Age in years as well as sex was ascertained by UK Biobank at baseline assessment. The Townsend deprivation index was used in the current study as an indicator of material deprivation and was assigned to each participant corresponding to their residential postcode at recruitment [34]. Educational qualifications, ethnicity, household income, and smoking status were captured on the touchscreen questionnaire. Education was divided into primary, secondary, post-secondary non-tertiary, and tertiary education. Ethnicity was categorized into white and non-white because 95% of the study participants were of white ethnicity. Household income (GBP) was categorized into < 18,000, 18,000–30,999, 31,000–51,999, 52,000–100,000, and > 100,000 categories. Smoking status was categorized into never, former, and current smoker. Standard alcohol units (alcohol by volume equivalents) were derived from touchscreen questions on the number of typical volume drinks for each type of alcohol consumed per week. Diabetes and hypertension were derived from touchscreen and verbal interview questions on doctor-diagnosed conditions and medication usage. The definition of hypertension also incorporated measured systolic blood pressure ≥ 140 mm of mercury (mmHg) or diastolic blood pressure ≥ 90 mmHg. Body mass index (BMI; kg/m^2^) was derived from weight (kg) using scales and standing height (meters) measured during the physical examination. In order to capture geographical variability in diagnosis, country of residence was defined as the location of assessment centre attended at baseline. APOE genotype and a non-APOE dementia polygenic risk score (PRS) were derived from the genome-wide genotyping and imputation performed by UK Biobank [35]. APOE-ε4 status was derived using the APOE single nucleotide polymorphisms (SNPs) rs429358 and rs7412. The non-APOE dementia PRS was previously developed by Ebenau et al. [36] and after quality control was performed consisted of 38 SNPs. The dementia PRS was split into quintiles, and further categorized into ‘low’ (quintile 1), ‘intermediate’ (quintiles 2–4), and ‘high’ (quintile 5) groups, with a higher PRS indicating a greater dementia risk. More information on the derivation of the PRS can be found elsewhere [33].

### Statistical analysis

Cox proportional-hazards models using time on study as the underlying time-scale were used to estimate the association between eGFRcr-cys and incident dementia. eGFRcr-cys was entered into the main analyses as a three-group categorical variable: ≥ 90 (reference group), 60–89, and < 60 ml/min/1.73m^2^. Time on study in years was calculated from the date of attending baseline assessment until the date of first incident dementia diagnosis, date of death, date of loss to follow-up, or end of follow-up, whichever occurred first. The end of follow-up was based on the availability of the medical record data in UK Biobank, which was censored on 30^th^ September 2021 for England, 31^st^ July 2021 for Scotland, and 28^th^ February 2018 for Wales. The proportional hazards assumption was visually assessed using scaled Schoenfeld residuals. There was no evidence that any of the variables included in the analyses violated the proportional hazards assumption. All analyses were first minimally adjusted for age in years and sex, and then additionally adjusted in the main model for ethnicity, Townsend deprivation index (quintiles), education, household income in GBP, country, smoking status, weekly alcohol intake in units, BMI (< 18.5, 18.5- < 25, 25- < 30, ≥ 30), diabetes (no, yes), hypertension (no, yes), and APOE status (*ε3/ε3*, *ε4/ε4* or *ε3/ε4*, *ε2/ε2* or *ε3/ε2*). Participants with missing data or who responded “prefer not to answer/do not know” for any of the covariates were assigned to a separate category for that covariate. In order to investigate whether this missingness could bias the results, a sensitivity analysis was conducted whereby multiple imputation by chained equations with 100 imputations was used to impute missing covariate values. Individuals with missing data for the exposure (*n* = 14,314) were excluded from the analysis. Because reduced physical activity was observed among patients with poor kidney function, in another sensitivity analysis, we further adjusted for physical activity, which could be conceptualised as either a confounder or mediator in the relationship between kidney function and dementia risk. Physical activity was assessed using questions adapted for the touchscreen questionnaire from the validated short International Physical Activity Questionnaire [37]. The time spent in vigorous, moderate, and walking activity was weighted by the energy expended for these categories of activity, to produce the total metabolic equivalent of task (MET) minutes per week (≤ 1200, > 1200). We conducted one other sensitivity analysis where the models were restricted to different follow-up periods: < 5 years, 5–10 years, and ≥ 10 years. This was to examine for potential reverse causation whereby the prodromal stages of dementia could affect kidney function years prior to a clinical dementia diagnosis [38]. In this scenario, the strength of associations would be stronger in the early years of follow-up and attenuate or become null in later years. We investigated the associations between eGFR and the risk of dementia with cubic splines, and the knots were set every 15 mL/min/1.73m^2^ of eGFR. In a secondary analysis, we expanded the < 60 ml/min/1.73m^2^ eGFRcr-cys category into 45–59, 30–44, and < 30 ml/min/1.73m^2^ to investigate whether a dose–response association was observed with worsening kidney function. We repeated the main analysis and the sensitivity analysis using different follow-up periods with the exposure entered into the model with an additional category of ≥ 105 ml/min/1.73m^2^. This was performed as previous studies have found evidence of an increased risk of dementia at the high end of eGFR status [6, 39].

The association between albuminuria and dementia was investigated by entering ACR into the model as the main exposure. Cubic splines were also used to explore the associations between ACR and dementia risk, and the knots were fixed at 3, 30, 100, and 200 mg/mmol. As eGFR and ACR are typically used together to determine the severity of CKD, we investigated the joint effect of each eGFRcr-cys and ACR group in association with incident dementia.

In further secondary analyses, the interactions between eGFRcr-cys and sociodemographic and genetic factors were investigated by entering interaction terms for eGFRcr-cys x age (60–64, ≥ 65 years old), eGFRcr-cys x sex, eGFRcr-cys x non-APOE PRS, and eGFRcr-cys x APOE status into separate models. Models that included the non-APOE dementia PRS were also adjusted for the first 10 principal components of ancestry. Effect estimates within each stratum of age, sex, non-APOE PRS, and APOE were obtained for eGFRcr-cys. We also investigated whether different ways of measuring eGFR status altered the findings by repeating the main analyses using eGFRcr and eGFRcys as the exposures. Finally, to investigate whether stroke could be driving any relationship between eGFRcr-cys and dementia as explored in previous studies [16, 19], we repeated the main analysis censoring for incident stroke (based on ICD-10 I60-64 codes). This study is reported following the Strengthening the Reporting of Observational Studies in Epidemiology (STROBE) reporting guideline. All p-values were two-sided, with statistical significance set at < 0.05. Analyses were performed using STATA/MP version 17 (StataCorp, College Station, TX, USA) and R version 4.0.2 (R Foundation for Statistical Computing, Vienna, Austria).

## Results

Of 217,465 participants aged 60 years or older at baseline, 166 with prevalent dementia, 283 with prevalent end-stage kidney disease, and 14,314 with missing eGFRcr-cys were excluded, resulting in a final sample of 202,702 participants. Of these, 64,082 (31.6%), 129,095 (63.7%) and 9,525 (4.7%) participants were classified as eGFRcr-cys ≥ 90, 60–89 and < 60 ml/min/1.73m^2^, respectively. Table 1 provides an overview of baseline characteristics by eGFRcr-cys status.

**Table 1:** Baseline characteristics of 202,716 participants by eGFRcr-cys status

	**eGFRcr-cys ml per min per 1.73**^**2**^		
**Characteristic**	** < 60** ***N* = 9,525**	**60–89** ***N* = 129,095**	** ≥ 90** ***N* = 64,082**
Age in years, mean (SD)	65.4 (2.8)	64.4 (2.9)	63.4 (2.7)
Women, N (%)	5,041 (52.9)	67,640 (52.4)	33,994 (53.1)
Ethnicity, N (%)			
White	9,085 (95.9)	124,852 (97.2)	61,980 (97.1)
Non-white	387 (4.1)	3,626 (2.8)	1,835 (2.9)
Townsend deprivation index, quintiles, N (%)			
1 (least deprived)	1,470 (15.4)	25,386 (19.7)	13,736 (21.5)
2	1,638 (17.2)	25,635 (19.9)	13,166 (20.6)
3	1,778 (18.7)	25,778 (20.0)	12,938 (20.2)
4	1,937 (20.3)	26,009 (20.2)	12,556 (19.6)
5 (most deprived)	2,698 (28.3)	26,177 (20.3)	11,631 (18.2)
Education, N (%)			
Primary	3,690 (39.5)	36,356 (28.6)	14,247 (22.5)
Secondary	3,422 (36.6)	55,577 (43.7)	30,297 (47.8)
Post-secondary non-tertiary	909 (9.7)	13,733 (10.8)	7,137 (11.3)
Tertiary	1,333 (14.3)	21,545 (16.9)	11,696 (18.5)
Household income in GBP, N (%)			
< 18,000	3,642 (49.3)	37,259 (35.8)	15,005 (28.4)
18,000–30,999	2,288 (31.0)	34,104 (32.7)	17,276 (32.7)
31,000–51,999	1,011 (13.7)	20,792 (20.0)	12,485 (23.6)
52,000–100,000	382 (5.2)	9,780 (9.4)	6,450 (12.2)
> 100,000	63 (0.9)	2,244 (2.2)	1,631 (3.1)
Country, N (%)			
England	8,426 (88.5)	115,219 (89.3)	57,084 (89.1)
Scotland	683 (7.2)	8,732 (6.8)	4,547 (7.1)
Wales	416 (4.4)	5,144 (4.0)	2,451 (3.8)
Smoking status, N (%)			
Never	4,152 (44.0)	63,886 (49.8)	32,640 (51.2)
Former	4,204 (44.5)	53,127 (41.4)	26,913 (42.2)
Current	1,084 (11.5)	11,273 (8.8)	4,198 (6.6)
Weekly alcohol intake in units, mean (SD)	7.8 (11.0)	10.7 (11.8)	13.0 (12.5)
BMI, N (%)			
Underweight	26 (0.3)	406 (0.3)	438 (0.7)
Normal	1,440 (15.2)	32,552 (25.3)	25,428 (39.8)
Overweight	3,663 (38.7)	59,684 (46.4)	27,982 (43.8)
Obese	4,330 (45.8)	35,964 (28.0)	9,991 (15.7)
Diabetes, N (%)			
No	7,769 (81.6)	120,546 (93.38)	59.846 (93.39)
Yes	1,756 (18.4)	8,549 (6.6)	4,236 (6.6)
Hypertension, N (%)			
No	1,100 (11.9)	26,334 (22.1)	14,900 (25.9)
Yes	8,166 (88.1)	92,664 (77.9)	42,706 (74.1)
Physical activity			
Low (≦1200 MET minutes per week)	3,208 (45.8)	35,462 (35.1)	16,199 (31.4)
High (> 1200 MET minutes per week)	3,804 (54.3)	65,658 (64.9)	35,379 (68.6)
APOE alleles, N (%)			
*ε3/ε3*	5,657 (61.2)	75,296 (60.4)	37,092 (60.0)
*ε4/ε4* or *ε3/ε4*	2,227 (24.2)	32,298 (25.9)	16,962 (27.5)
*ε2/ε2* or* ε3/ε2*	1,306 (14.2)	16,984 (13.6)	7,721 (12.5)
Non-APOE dementia PRS, N (%)			
Low	1,521 (19.7)	21,127 (20.0)	10,607 (20.1)
Intermediate	4,663 (60.5)	63,585 (60.1)	31,517 (59.7)
High	1,526 (19.8)	21,087 (19.9)	10,641 (20.2)

*Abbreviations*: *APOE* Apolipoprotein E, *BMI* Body Mass Index, *eGFRcr-cys* Estimated Glomerular Filtration Rate Creatinine-Cystatin C Equation, *GBP* British Pound Sterling, *MET* Metabolic Equivalent of Task, *N* Number of participants, *PRS* Polygenic Risk Score, *SD* Standard Deviation

**Fig. 1:**
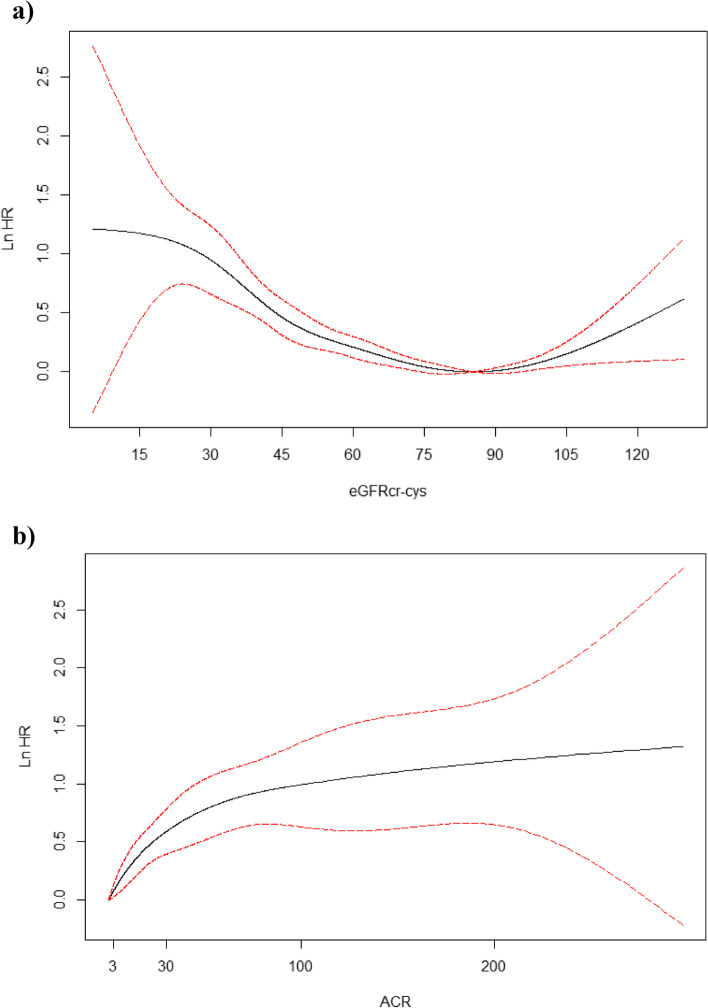
Smoothing spline plots showing the log Hazard Ratio for incident dementia by **a**) eGFRcr-cys ml/min/1.73m^2^ and **b**) ACR mg/mmol. 95% confidence intervals shown as red dashed lines. Abbreviations: ACR, Albumin-to-Creatinine Ratio, eGFRcr-cys, Estimated Glomerular Filtration Rate Creatinine-Cystatin C Equation. Models adjusted for age, sex, ethnicity, Townsend deprivation index, education, household income, country, smoking status, alcohol intake, body mass index, hypertension, diabetes, and APOE status

Over 15 years of follow-up (mean = 11.8 years, Standard Deviation = 2.2), 6,042 participants were diagnosed with incident dementia. In age and sex-adjusted models, the Hazard Ratios (HR) were 1.01 (95% Confidence Interval [CI] 0.95–1.07) and 1.62 (95% CI 1.46–1.79) for eGFRcr-cys 60–89 and < 60 ml/min/1.73m^2^, respectively, compared to ≥ 90 ml/min/1.73m^2^ (Table 2). In fully adjusted models, the direction of associations remained consistent although the strength was attenuated in those with eGFRcr-cys < 60 ml/min/1.73m^2^ (HR = 1.42, 95% CI 1.28–1.58). Table S2 demonstrates the effect of incremental adjustment and individual inclusion of each covariate to the age and sex-adjusted model. Additional adjustment for physical activity did not alter the findings: HR = 1.00 (95% CI 0.94–1.06) and 1.41 (95% CI 1.27–1.56) for eGFRcr-cys 60–89 and < 60 ml/min/1.73m^2^, respectively, compared to ≥ 90 ml/min/1.73m^2^; higher level of physical activity was associated with a lower risk of dementia (HR = 0.87, 95% CI 0.82–0.9). The results also remained similar after using multiple imputation for missing covariate data: HR = 1.00 (95% CI 0.94–1.06) and 1.43 (95% CI 1.28–1.58) for eGFRcr-cys 60–89 and < 60 ml/min/1.73m^2^, respectively, compared to ≥ 90 ml/min/1.73m^2^. When restricting the models to different periods of follow-up to investigate for potential reverse causation, the associations remained similar to the main findings (Table 2). A smoothing spline plot shows the risk of incident dementia increases as eGFRcr-cys decreases (Fig. 1a). This dose–response association between worsening kidney function and incident all-cause dementia was also observed when expanding eGFRcr-cys into Kidney Disease: Improving Global Outcomes (KDIGO) categories [17] for grading risk of chronic kidney disease, with HRs of 1.34 (95% CI 1.20–1.51), 1.59 (95% CI 1.27–1.99) and 2.93 (95% CI 2.02–4.24) for 45–59, 30–44 and < 30 ml/min/1.73m^2^, respectively, compared to ≥ 90 ml/min/1.73m^2^.

**Table 2:** Cox proportional-hazards models investigating the association between eGFRcr-cys status and incident all-cause dementia by different follow-up periods

		**Age and sex adjusted**	**Fully adjusted**^**a**^
**Follow-up period**	**Cases/Population**	**HR (95% CI)**	**HR (95% CI)**
**Complete follow-up**			
≥ 90	1,618/64,082	1 (Reference)	1 (Reference)
60–89	3,932/129,095	1.01 (0.95–1.07)	1.00 (0.94–1.06)
< 60	492/9,525	1.62 (1.46–1.79)	1.42 (1.28–1.58)
** ≤ 5 years**			
≥ 90	149/64,085	1 (Reference)	1 (Reference)
60–89	361/129,095	0.97 (0.80–1.18)	0.98 (0.80–1.19)
< 60	51/9,525	1.59 (1.15–2.20)	1.36 (0.98–1.90)
** > 5 to 10 years**			
≥ 90	717/62,661	1 (Reference)	1 (Reference)
60–89	1,731/125,140	0.99 (0.91–1.08)	1.00 (0.91–1.09)
< 60	240/8,792	1.71 (1.48–1.99)	1.53 (1.31–1.78)
** > 10 years**			
≥ 90	752/58,436	1 (Reference)	1 (Reference)
60–89	1,840/114,968	1.03 (0.94–1.12)	1.01 (0.93–1.11)
< 60	201/7,382	1.52 (1.30–1.78)	1.33 (1.13–1.56)

*Abbreviations*: *CI* Confidence Interval, *eGFRcr-cys* Estimated Glomerular Filtration Rate Creatinine-Cystatin C Equation, *HR* Hazard Ratio

^a^Models adjusted for age, sex, ethnicity, Townsend deprivation index, education, household income, country, smoking status, alcohol intake, body mass index, hypertension, diabetes, and APOE status

The spline also shows an increased risk of incident dementia at higher levels of eGFRcr-cys (Fig. 1a). We investigated this further and found that an eGFRcr-cys of ≥ 105 ml/min/1.73m^2^ was associated with an increased risk of incident dementia (HR = 1.34, 95% CI 1.13–1.60), compared to an eGFRcr-cys of 90–104 ml/min/1.73m^2^. However, the associations were strongest when restricted to ≤ 5 years follow-up (HR = 2.32, 95% CI 1.47–3.66), then substantially attenuated when restricted to 5–10 years follow-up (HR = 1.41, 95% CI 1.09–1.82), and were null when restricted to > 10 years follow-up (HR = 1.08, 95% CI 0.81–1.44).

We repeated the analyses using ACR as the main exposure for kidney function. In fully adjusted models, the HRs for incident dementia were 1.16 (95% CI 1.07–1.26) and 2.24 (95% CI 1.79–2.80) for moderately (3–30 mg/mmol) and severely (> 30 mg/mmol) increased ACR, respectively, compared to normal or mildly increased ACR (< 3 mg/mmol, reference group). A smoothing spline plot shows a dose–response association between ACR and incident dementia, which plateaus around an ACR of 80 mg/mmol (Fig. 1b). However, it should be noted that only 0.2% of participants had an ACR of 80 mg/mmol or above. When combining eGFRcr-cys and ACR status, the risk of incident dementia correspondingly increased as kidney function based on either measure decreased (Table 3). For instance, amongst participants with severely decreased eGFRcr-cys, the HRs were 2.46 (95% CI 1.32–4.60), 3.23 (95% CI 1.61–6.48), and 4.70 (95% CI 2.34–9.43) for normal, moderate and severe ACR, respectively, compared to participants with both normal eGFRcr-cys and normal ACR. The interaction between eGFRcr-cys and ACR was not statistically significant (*p*-value = 0.89).

**Table 3:**
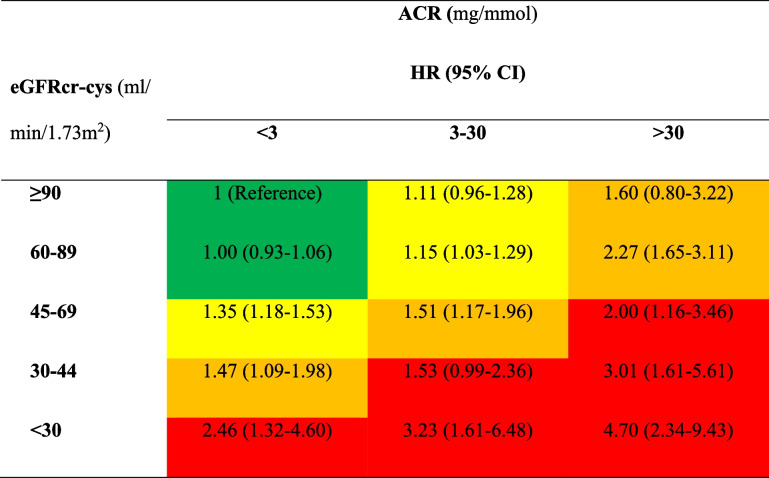
Cox proportional-hazards models investigating the association between combined eGFRcr-cys and ACR status with risk of incident all-cause dementia

*Abbreviations*: *ACR* Albumin-to-Creatinine Ratio, *CI* Confidence Interval, *eGFRcr-cys* Estimated Glomerular Filtration Rate Creatinine-Cystatin C Equation, *HR* Hazard Ratio

Model adjusted for age, sex, ethnicity, Townsend deprivation index, education, household income, country, smoking status, alcohol intake, body mass index, hypertension, diabetes, and APOE status

(Colors represent the risk categories of CKD according to 2012 KDIGO classification. Green: low risk; Yellow: moderately increased risk; Orange: high risk; Red: very high risk)

In secondary analyses, we found evidence of a statistically significant interaction between eGFRcr-cys status and APOE with risk of incident dementia, but not for age, sex, or non-APOE dementia PRS (Table 4, Table S3). When stratifying by APOE status, the associations were weaker amongst those with a higher genetic risk of dementia (*ε4/ε4 or ε3/ε4*), and stronger amongst those with a normal (*ε3/ε3*) and lower (*ε2/ε2 or ε3/ε2*) genetic risk of dementia (Table 4). For the *ε2/ε2 or ε3/ε2* group, a dose–response association was observed with incident dementia, with HRs of 1.28 (95% CI 1.00–1.64) and 2.25 (95% CI 1.58–3.20) for eGFRcr-cys 60–89 and < 60 ml/min/1.73m^2^, respectively, compared to ≥ 90 ml/min/1.73m^2^. The associations remained similar to the main findings when entering eGFRcr or eGFRcys as the exposure to the fully adjusted models (Fig. S1a and b, Table S4). The findings also remained similar when censoring for stroke, with HRs of 0.99 (95% CI 0.93–1.05) and 1.33 (95% 1.19–1.50) observed for eGFRcr-cys 60–89 and < 60 ml/min/1.73m^2^, respectively, compared to ≥ 90 ml/min/1.73m^2^.

**Table 4:** Cox proportional-hazards models investigating the association between eGFRcr-cys and incident all-cause dementia by genetic risk for dementia

Genetic risk for dementia	Cases/Population	Age and sex adjusted HR (95% CI)	*p*-value for interaction	Fully adjusted^a^ HR (95% CI)	*p*-value for interaction
**Dementia non-APOE PRS**					
*Low*					
≥ 90	184/10,607	1 (Reference)		1 (Reference)	
60–89	491/21,127	1.11 (0.94–1.32)		1.07 (0.90–1.27)	
< 60	58/1,521	1.72 (1.28–2.31)		1.43 (1.06–1.92)	
*Intermediate*					
≥ 90	762/31,517	1 (Reference)		1 (Reference)	
60–89	1,846/63,585	1.01 (0.92–1.10)		1.00 (0.92–1.09)	
< 60	259/4,663	1.80 (1.56–2.08)		1.58 (1.37–1.83)	
*High*					
≥ 90	341/10,641	1 (Reference)		1 (Reference)	
60–89	877/21,087	1.07 (0.95–1.22)		1.08 (0.95–1.23)	
< 60	95/1,526	1.60 (1.27–2.01)	0.48	1.39 (1.10–1.74)	0.42
**APOE allele**					
*ε3/ε3*					
≥ 90	595/37,092	1 (Reference)		1 (Reference)	
60–89	1,494/75,296	1.02 (0.93–1.13)		1.00 (0.90–1.10)	
< 60	211/5,657	1.81 (1.54–2.12)		1.50 (1.28–1.76)	
*ε4/ε4 or ε3/ε4*					
≥ 90	874/16,962	1 (Reference)		1 (Reference)	
60–89	1,990/32,298	1.01 (0.93–1.09)		0.98 (0.91–1.07)	
< 60	215/2,227	1.53 (1.32–1.78)		1.27 (1.09–1.48)	
*ε2/ε2 or ε3/ε2*					
≥ 90	83/7,721	1 (Reference)		1 (Reference)	
60–89	288/16,984	1.32 (1.03–1.68)		1.28 (1.00–1.64)	
< 60	49/1,306	2.71 (1.90–3.86)	0.04	2.25 (1.58–3.20)	0.04

*Abbreviations*: *APOE* Apolipoprotein E, *CI* Confidence Interval, *eGFRcr-cys* Estimated Glomerular Filtration Rate Creatinine-Cystatin C Equation, *HR* Hazard Ratio, *PRS* Polygenic Risk Score

^a^Models adjusted for age, sex, ethnicity, Townsend deprivation index, education, household income, country, smoking status, alcohol intake, body mass index, hypertension, and diabetes. The dementia non-APOE PRS analyses were also adjusted for APOE and the first 10 principal components of ancestry

## Discussion

In this large, population-based cohort, we found that decreased kidney function and increased albuminuria were associated with a higher risk of dementia over 15 years of follow-up. These associations persisted when accounting for potential reverse causation by restricting to dementia cases diagnosed 10 years after kidney function was measured. We also found a strong dose–response association with incident dementia when combining both eGFR and ACR to classify the severity of kidney function. Furthermore, the associations were much stronger amongst participants with a lower genetic risk of dementia based on APOE status, but non-APOE PRS did not modify the associations.

The prior literature on the association between eGFR and dementia risk is inconclusive. One meta-analysis [13], which included 8 prospective population-based studies examining the association between eGFR and cognitive impairment or dementia, concluded that the overall association did not reach statistical significance (odds ratio [95% CI] for eGFR < 60 ml/min/1.73m^2^ = 1.28 [0.99–1.65]); among the three studies that focused on dementia risk, the largest had 7,839 participants (564 incident dementia cases) [40], and the longest had a median follow-up duration of 6 years [41]. Since the meta-analysis, other longitudinal studies have been published. The HUNT study [42] and the Hisayama study [43] found no association between eGFR and dementia, whilst the Stockholm Creatinine Measurements (SCREAM) project [6], the IMRD-THIN database [5], and the Whitehall II cohort study [19] found worse eGFR was associated with higher dementia risk. Most aforementioned studies estimated the eGFR based on serum creatinine alone. The few studies that calculated eGFR based on various biomarkers reported conflicting results. The Atherosclerosis Risk in Communities (ARIC) study reported that eGFRcr was not associated with dementia, whereas worse eGFRcys was positively associated with dementia, and eGFRcr-cys was only positively associated with dementia when measured at an older age (4,626 participants, 438 incident dementia cases) but not at midlife (9,967 participants, 1821 incident dementia cases) [14]. The Shanghai Aging Study (1,412 participants, 113 incident dementia cases) observed a positive association between worse eGFRcr-cys and dementia, but results for eGFRcr and eGFRcys were not significant [15]. In contrast, the Rotterdam study, with 5,993 participants (758 incident dementia cases) and 11.6 years of follow-up, found positive associations between eGFRcr, eGFRcys, and eGFRcr-cys only with stroke but not with dementia [16]. Our findings were consistent with previous studies that found a positive association between poor kidney functionand risk of dementia and we have demonstrated that the association between eGFR and dementia was robust whether using the creatinine equation (CKD-EPI 2009), cystatin C equation (CKD-EPI 2012), or creatinine–cystatin C equation (CKD-EPI 2012), which is comparable to a recent study by Wu et al. [44] using data from the UK Biobank. Importantly, with the follow-up duration of 15 years, we further found that these associations were robust when restricting to participants with 5–10 as well as > 10 years of follow-up to account for reverse causation, and also when censoring for stroke.

We also found that eGFR above 105 ml/min/1.73m^2^ was associated with an increased risk of dementia, a finding consistent with the results from the SCREAM project [6]. Similar U-shape relationships have been observed with other health outcomes, including total mortality [45], cardiovascular mortality [45], coronary artery calcification [46], and cognitive function [47]. Upon further interrogation, we found that an eGFR ≥ 105 ml/min/1.73m^2^ was associated with more than double the risk of dementia within five years of follow-up, but was not associated after ten years of follow-up. This substantial attenuation provides strong evidence that the association at the higher end of eGFR is likely due to reverse causation. Those with eGFR ≥ 105 ml/min/1.73m^2^ might be comprised of various heterogeneous populations that all contributed to the higher dementia risk, such as individuals with diabetes or a pre-diabetic state [48] or people with cachexia, in whom eGFR is overestimated due to reduced muscle mass. [49].

The dose–response association that we observed for ACR, the preferred method for quantifying albuminuria [17], was consistent with the existing literature [5, 13, 42, 50]. Albuminuria is the result of serum protein leakage into the urine and is an important marker of glomerular pathologies, microcirculatory damage, and systemic endothelial dysfunction [51]. The brain and the kidney share similar microvascular structures; therefore, both have similar responses to diseases and are sensitive to endothelial dysfunction [52]. The presence of albuminuria may reflect simultaneous protein leakage through the blood–brain barrier, appearing as white matter hyperintensities on magnetic resonance imaging of the brain [52], which were causally linked to dementia [53]. The KDIGO 2012 guideline classifies CKD using the combination of both GFR and ACR. We followed such classifications and categorized our cohort based on both of these measures and found that they were strongly associated with a higher risk of dementia. These findings suggested that the use of a combination of these two measures may provide additional information when evaluating the possible risk of dementia for patients with kidney dysfunction.

Other mechanistic pathways underlying the association between kidney dysfunction and dementia have been proposed. These include increased uremic neurotoxins, decreased kidney neurotrophins, and uremia neuroinflammation [54, 55]. In addition, genetic factors may play a role in the pathogenesis of cognitive dysfunction in CKD [54]. We found that APOE status significantly interacted with kidney function to modify the risk of dementia, with the associations strongest in those with a lower genetic risk of dementia and weaker in those with a higher genetic risk of dementia. There is evidence that APOE *ε2* increases the rate of kidney disease progression compared to other APOE alleles, which could consequently accelerate the likelihood of developing dementia in this group [20]. Alternatively, the increased relative association could be due to the lower baseline risk of dementia in those with APOE *ε2* allele. In contrast, we found no evidence of an interaction effect between non-APOE PRS and kidney function with risk of dementia. The strength of associations were similar regardless of non-APOE genetic risk with no evidence that a higher risk attenuated associations.

The current study has several strengths. Various kidney-related biomarkers were measured at baseline, which enabled us to explore the associations between eGFR calculated from three different CKD-EPI equations and the combined categories based on both eGFR and ACR with dementia risk. The large sample size, detailed data collection, and the long follow-up enabled us to investigate several important aspects of the relationship between kidney function and dementia, such as reverse causation and interaction with genetic factors.

The current study also has several limitations. First, the diagnosis of dementia was based on hospital inpatient and death registry data, which although valid for capturing all-cause dementia, does not reliably discriminate between specific dementia subtypes [30]. Second, the UK Biobank cohort is not completely representative of the general population because participants are generally healthier and with higher socioeconomic status [56]. Third, we only had a single measure of kidney function and albuminuria, so were unable to explore the change in these over time with the risk of dementia. Fourth, we do not have adequate information on treatments for CKD, such as dialysis. Finally, due to the observational nature of the study, we are unable to determine causality or completely rule out the potential for residual confounding.

In conclusion, results from the current large prospective cohort study showed that both worsening kidney function and albuminuria were associated with a higher risk of dementia. Furthermore, the significant synergistic effect suggested that the use of eGFR in conjunction with ACR may perform even better for dementia risk stratification. Our findings provide additional evidence for the role of kidney dysfunction as a modifiable risk factor for dementia, and also highlight the possibility that genetic risk factors for dementia modify the association.

### Supplementary Information

**Additional file 1.** 

## Data Availability

Researchers can apply to UK Biobank to access the data used in this study (https://ukbiobank.ac.uk/).
