# Supplementary material for: Kidney function, albuminuria, and their modification by genetic factors and risk of incident dementia in UK Biobank
Source: Alzheimers Res Ther. 2023 Aug 21;15:138. doi: 10.1186/s13195-023-01248-z (PMC10440913; doi:10.1186/s13195-023-01248-z)
Supplement: Supplementary file 1 — Additional file 1. [file 13195_2023_1248_MOESM1_ESM.docx]

**Table S1 -** International Classification of Disease codes used to ascertain dementia

| **ICD-9** | **ICD-10** |
| --- | --- |
| 331.0, 290.4, 331.1, 290.2, 290.3, 291.2, 294.1, 331.2, 331.5 | F00, F00.0, F00.1, F00.2, F00.9, G30, G30.0, G30.1, G30.8, G30.9, F01, F01.0, F01.1, F01.2, F01.3, F01.8, F01.9, I67.3, F02.0, G31.0, A81.0, F02, F02.1, F02.2, F02.3, F02.4, F02.8, F03, F05.1, F10.6, G31.1, G31.8 |

Abbreviations: ICD, International Classification of Disease

**Table S2 –** Effect of adjustment for covariates on the association between eGFRcr-crcys and risk of incident all-cause dementia

|  | **eGFRcr-cys ml per min per 1.73m^2^** | | |  |
| --- | --- | --- | --- | --- |
| **Adjustments** | **≥90**  **HR (95% CI)** | **60-89**  **HR (95% CI)** | **<60**  **HR (95% CI)** | **x^2^ (p-value)** |
| *Incremental adjustment* |  |  |  |  |
| age and sex | 1 (Reference) | 1.01 (0.95-1.07) | 1.62 (1.46-1.79) |  |
| + ethnicity | 1 (Reference) | 1.01 (0.95-1.07) | 1.62 (1.46-1.79) | 13.9 (0.001) |
| + Townsend index | 1 (Reference) | 1.00 (0.94-1.06) | 1.56 (1.40-1.72) | 175.7 (<0.001) |
| + education | 1 (Reference) | 0.98 (0.93-1.04) | 1.50 (1.36-1.67) | 119.7 (<0.001) |
| + income | 1 (Reference) | 0.97 (0.91-1.03) | 1.45 (1.31-1.61) | 247.3 (0.008) |
| + country | 1 (Reference) | 0.97 (0.91-1.03) | 1.45 (1.31-1.61) | 30.8 (<0.001) |
| + smoking status | 1 (Reference) | 0.96 (0.91-1.02) | 1.44 (1.29-1.59) | 36.0 (<0.001) |
| + alcohol | 1 (Reference) | 0.95 (0.89-1.01) | 1.39 (1.25-1.54) | 40.5 (<0.001) |
| + BMI | 1 (Reference) | 0.96 (0.90-1.02) | 1.40 (1.26-1.56) | 39.6 (<0.001) |
| + diabetes | 1 (Reference) | 0.98 (0.92-1.04) | 1.35 (1.22-1.51) | 268.6 (<0.001) |
| + hypertension | 1 (Reference) | 0.98 (0.92-1.04) | 1.36 (1.22-1.51) | 0.72 (0.40) |
| + APOE status | 1 (Reference) | 1.00 (0.94-1.06) | 1.42 (1.28-1.58) | 2009.0 (<0.001) |
| *Age and sex adjusted models with separate inclusion of each covariate* | | | | |
| ethnicity | 1 (Reference) | 1.01 (0.95-1.07) | 1.62 (1.46-1.79) | 13.9 (0.001) |
| Townsend index | 1 (Reference) | 1.00 (0.94-1.06) | 1.56 (1.41-1.73) | 183.9 (<0.001) |
| education | 1 (Reference) | 0.99 (0.93-1.05) | 1.55 (1.40-1.71) | 166.7 (<0.001) |
| income | 1 (Reference) | 0.98 (0.92-1.04) | 1.51 (1.36-1.67) | 411.4 (<0.001) |
| country | 1 (Reference) | 1.01 (0.95-1.07) | 1.62 (1.46-1.80) | 31.9 (<0.001) |
| smoking status | 1 (Reference) | 1.00 (0.94-1.06) | 1.58 (1.43-1.75) | 79.4 (<0.001) |
| alcohol | 1 (Reference) | 0.98 (0.93-1.04) | 1.55 (1.40-1.72) | 68.7 (<0.001) |
| BMI | 1 (Reference) | 1.01 (0.95-1.07) | 1.59 (1.43-1.77) | 53.4 (<0.001) |
| diabetes | 1 (Reference) | 1.01 (0.95-1.07) | 1.49 (1.35-1.65) | 337.7 (<0.001) |
| hypertension | 1 (Reference) | 1.01 (0.95-1.07) | 1.62 (1.46-1.80) | 1.6 (0.21) |
| APOE status | 1 (Reference) | 1.03 (0.97-1.09) | 1.71 (1.55-1.90) | 2012.8 (<0.001) |

Abbreviations: CI, Confidence Interval, eGFRcr-cys, Estimated Glomerular Filtration Rate Creatinine-Cystatin C Equation, HR, Hazard Ratio

**Table S3 -** Cox proportional-hazards models investigating the association between eGFRcr-crys and risk of incident all-cause dementia by age and sex

| **Characteristic** |  | **Age and sex-adjusted**  **HR (95% CI)** | **p-value for interaction** | **Fully adjusted^*^**  **HR (95% CI)** | **p-value for interaction** |
| --- | --- | --- | --- | --- | --- |
|  | **Cases/Population** |  |  |  |  |
| **Age** |  |  |  |  |  |
| *<65 years* |  |  |  |  |  |
| ≥90 | 680/43,048 | 1 (Reference) |  | 1 (Reference) |  |
| 60-89 | 1,167/66,822 | 1.12 (1.02-1.24) |  | 1.11 (1.01-1.22) |  |
| <60 | 110/3,452 | 2.29 (1.88-2.81) |  | 1.84 (1.50-2.25) |  |
| *≥65 years* |  |  |  |  |  |
| ≥90 | 938/21,034 | 1 (Reference) |  | 1 (Reference) |  |
| 60-89 | 2,765/62,273 | 1.01 (0.94-1.09) |  | 1.02 (0.91-1.06) |  |
| <60 | 382/6,073 | 1.64 (1.45-1.84) | 0.02 | 1.38 (0.94-1.09) | 0.12 |
| **Sex** |  |  |  |  |  |
| *Female* |  |  |  |  |  |
| ≥90 | 763/33,994 | 1 (Reference) |  | 1 (Reference) |  |
| 60-89 | 1,882/67,640 | 1.03 (0.95-1.12) |  | 1.03 (0.94-1.12) |  |
| <60 | 249/5,041 | 1.67 (1.45-1.93) |  | 1.51 (1.30-1.75) |  |
| *Male* |  |  |  |  |  |
| ≥90 | 855/30,088 | 1 (Reference) |  | 1 (Reference) |  |
| 60-89 | 2,050/61,455 | 0.98 (0.91-1.07) |  | 0.98 (0.91-1.07) |  |
| <60 | 243/4,484 | 1.57 (1.36-1.82) | 0.68 | 1.35 (1.17-1.56) | 0.53 |

Abbreviations: CI, Confidence Interval, eGFRcr-cys, Estimated Glomerular Filtration Rate Creatinine-Cystatin C Equation, HR, Hazard Ratio

^*^ Models adjusted for age, sex, ethnicity, Townsend deprivation index, education, household income, country, smoking status, alcohol intake, body mass index, hypertension, diabetes, and APOE status

**Table S4 -** Cox proportional-hazards models investigating the association between different measures of eGFR and risk of incident all-cause dementia

|  |  | **Age and sex-adjusted** | **Fully adjusted^*^** |
| --- | --- | --- | --- |
| **eGFR measure, ml per min per 1.73m^2^** | **Cases/Population** | **HR (95% CI)** | **HR (95% CI)** |
| **eGFRcr** |  |  |  |
| ≥90 | 2,479/90,163 | 1 (Reference) | 1 (Reference) |
| 60-89 | 3,166/104,460 | 0.89 (0.84-0.94) | 0.93 (0.88-0.98) |
| <60 | 397/8,079 | 1.39 (1.24-1.54) | 1.31 (1.17-1.46) |
| **eGFRcys** |  |  |  |
| ≥90 | 1,396/56,476 | 1 (Reference) | 1 (Reference) |
| 60-89 | 3,792/128,960 | 1.01 (0.95-1.08) | 1.00 (0.94-1.06) |
| <60 | 854/17,266 | 1.62 (1.48-1.76) | 1.39 (1.27-1.53) |

Abbreviations: CI, Confidence Interval, eGFRcr, Estimated Glomerular Filtration Rate Creatinine Equation, eGFRcys, Estimated Glomerular Filtration Rate Cystatin C Equation, HR, Hazard Ratio

^*^ Models adjusted for age, sex, ethnicity, Townsend deprivation index, education, household income, country, smoking status, alcohol intake, body mass index, hypertension, diabetes, and APOE status

**Fig. S1 –** Smoothing spline plots showing the log Hazard Ratio for incident dementia by **a)** eGFRcr ml per min per 1.73m^2^ and **b)** eGFRcys ml per min per 1.73m^2^

**a)**

**
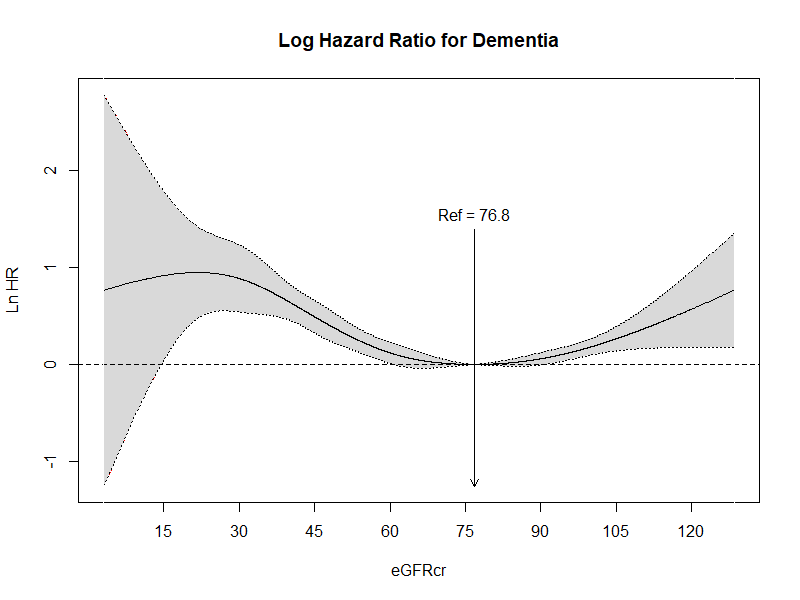
**

**b)**

**
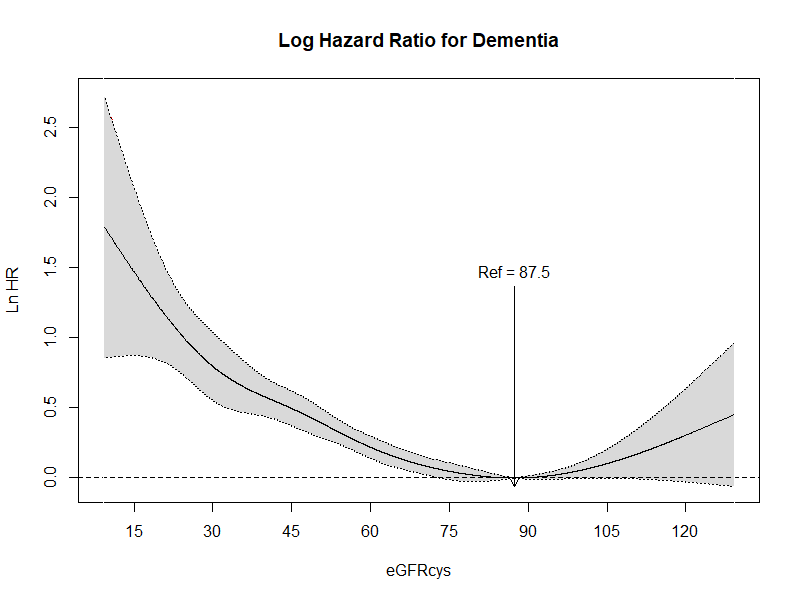
**

Models adjusted for age, sex, ethnicity, Townsend deprivation index, education, household income, country, smoking status, alcohol intake, body mass index, hypertension, diabetes, and APOE status
